# Threats to Internal Validity in Multiple-Baseline Design Variations

**DOI:** 10.1007/s40614-022-00326-1

**Published:** 2022-01-27

**Authors:** Timothy A. Slocum, Sarah E. Pinkelman, P. Raymond Joslyn, Beverly Nichols

**Affiliations:** Department of Special Education & Rehabilitation Counseling, Utah State University, 2865 Old Main Hill, Logan, UT 84322 USA

**Keywords:** Single-case design, Multiple baseline design, Concurrent, Nonconcurrent, Research methodology, Internal validity

## Abstract

Multiple baseline designs—both concurrent and nonconcurrent—are the predominant experimental design in modern applied behavior analytic research and are increasingly employed in other disciplines. In the past, there was significant controversy regarding the relative rigor of concurrent and nonconcurrent multiple baseline designs. The consensus in recent textbooks and methodological papers is that nonconcurrent designs are less rigorous than concurrent designs because of their presumed limited ability to address the threat of coincidental events (i.e., history). This skepticism of nonconcurrent designs stems from an emphasis on the importance of across-tier comparisons and relatively low importance placed on replicated within-tier comparisons for addressing threats to internal validity and establishing experimental control. In this article, we argue that the primary reliance on across-tier comparisons and the resulting deprecation of nonconcurrent designs are not well-justified. In this article, we first define multiple baseline designs, describe common threats to internal validity, and delineate the two bases for controlling these threats. Second, we briefly summarize historical methodological writing and current textbook treatment of these designs. Third, we explore how concurrent and nonconcurrent multiple baselines address each of the main threats to internal validity. Finally, we make recommendations for more rigorous use, reporting, and evaluation of multiple baseline designs.

Multiple baseline designs are the workhorses of single-case design (SCD) research and are the predominant design used in modern applied behavior analytic research (Coon & Rapp, 2018; Cooper et al., 2020). In a review of the SCD literature, Shadish and Sullivan (2011) found multiple baseline designs making up 79% of the SCD literature (54% multiple baseline alone, 25% mixed/combined designs). In addition, multiple baseline designs are increasingly used in literatures that are not explicitly behavior analytic. Smith (2012) found that SCD was reported in 143 different journals that span a variety of fields such as behavior analysis, psychology, education, speech, and pain management; across these fields, multiple baselines account for 69% of SCDs.

Given that multiple baseline designs make up such a large proportion of the existing SCD literature and current research activity, it is critical that SCD researchers thoroughly understand the specific ways that multiple baseline designs address potential threats to internal validity so that they can make experimental design decisions that optimize internal validity and accurately evaluate, discuss, and interpret the results of their research. Peer reviewers and editors who serve as gatekeepers for the scientific literature must also have a deep understanding of these issues so that they can distinguish between stronger and weaker research, ensure that information critical to evaluating internal validity is included in research reports, and assess the appropriateness of discussion and interpretation of results. Finally, practitioners whose work may be influenced by SCD research must understand these issues so they can give appropriate weight to research findings. A broad and general impression such as “these designs are relatively strong” is not sufficient to guide experimental design decisions or to evaluate particular variations of multiple baseline designs. Instead, a detailed understanding of how specific threats to internal validity are addressed in multiple baseline designs and specific design features that strengthen or weaken control for these threats are needed.

One area that has, in the past, been particularly controversial is the experimental rigor of concurrent versus nonconcurrent multiple baseline designs; that is, the degree to which each can rule out threats to internal validity. This controversy began soon after the first formal description of nonconcurrent multiple baseline designs by Hayes (1981) and Watson and Workman (1981). However, the specific issues in this controversy have never been thoroughly identified, discussed, and resolved; and instead a consensus emerged without the issues being explicitly addressed. This consensus is that nonconcurrent multiple baseline designs are substantially weaker than concurrent designs (e.g., Cooper et al., 2020; Johnston et al., 2020; Kazdin, 2021). Nonconcurrent designs are said to be substantially compromised with respect to internal validity and in general this limitation is ascribed to their supposed weakness in addressing threats of coincidental events (i.e., history). We challenge this assertion. A close examination of threats to internal validity in multiple baseline designs reveals and clarifies the critical design features that determine the degree of experimental control and internal validity of either type of multiple baseline. The purposes of this article are to (1) thoroughly examine the impact that threats to internal validity can have on concurrent and nonconcurrent multiple baseline designs; (2) describe the critical features of each design type that control for threats to internal validity; and (3) offer recommendations for use and reporting of concurrent and nonconcurrent multiple baseline designs.

## Basic Description of Multiple-baseline Designs and Threats to Internal Validity

It is surprising that there is no single consensus definition of multiple baseline designs. Textbooks commonly describe and characterize the design without clearly defining it. For the purposes of this article, we define a multiple baseline design as *a single-case experimental design that evaluates causal relations through the use of multiple baseline-treatment comparisons with phase changes that are offset in (1) real time (e.g., calendar date), (2) number of days in baseline, and (3) number of sessions in baseline*. (Our specification of phase change offset in terms of real time, days in baseline, and sessions in baseline is unusual. Reasons for these specifications will become clear later in the article.) These baseline-treatment comparisons, which we will refer to as “tiers,” differ from one another with respect to participants, behaviors, settings, stimulus materials, and/or other variables. *Concurrent multiple baseline designs are multiple baseline designs in which the tiers are synchronized in real time*. By *synchronized* we mean that “session 1” in all tiers takes place before “session 2” in any tier, and this ordinal invariance of session number across tiers is true for all sessions. So, for example, session 10 in tier 2 must take place at some time between tier 1’s session 9 and 11. *Nonconcurrent multiple baseline designs are those in which tiers are not synchronized in real time.* That is, session numbers do not necessarily correspond to the same periods of real time across tiers. For example, knowing the date of session 10 in tier 1 tells us nothing about the date of session 10 in tier 2.

Multiple baseline designs are intended to evaluate whether there is a functional (causal) relation between the introduction of the independent variable and changes in the dependent variable. A functional relation can be inferred if the pattern of data demonstrates experimental control—the experimenter’s ability to produce a change in the dependent variable in a precise and reliable fashion (Sidman, 1960). When determining whether a multiple baseline study demonstrates experimental control, researchers examine the data within and across tiers and also consider the extent to which alternative explanations (e.g., extraneous variables or confounds) could plausibly account for the obtained data patterns. If factors other than the experimenter’s manipulation of the independent variable could plausibly account for the obtained data patterns, experimental control has not been demonstrated and functional relations cannot be inferred. Research methodologists have identified numerous potential alternative explanations that are threats to internal validity (e.g., Campbell & Stanley, 1963; Cooper et al., 2020; Kazdin, 2021; Shadish et al., 2002). We will focus on the three types of threats that are addressed through comparisons between baseline and treatment phases in multiple baseline designs: maturation, testing and session experience, and coincidental events.^1^

*Maturation* refers to extraneous variables such physical growth, physiological changes, typical interactions with social and physical environments, academic instruction, and behavior management procedures that tend to cause changes in behavior over time (cf., Shadish et al., 2002). Maturational changes may be smooth and gradual, or they may be sudden and uneven. For example, physical growth and experiences with the environment can accumulate and result in relatively sudden behavioral changes when a toddler begins to walk. The key characteristic that maturational processes share is that they may produce behavioral changes that would be expected to accumulate as a *function of elapsed time* in the absence of participation in research.^2^ In order to control for maturation, we must attend to the passage of time—typically, calendar days.

*Testing and session experience* encompasses features of experimental sessions (both baseline and intervention phases) other than the independent variable that could cause changes in behavior. These could include presence of observers, testing procedures, exposure to testing stimuli, attention from implementers, being removed from the typical setting, exposure to a special setting, and so on. These variables share the key characteristic that their impact would be expected to accumulate as a *function of number of experimental sessions*. Control for testing and session experience requires attention to the number of sessions that participants experience.

*Coincidental events* (i.e., history) are specific events that occur at a particular time (or across a particular period) and could cause changes in behavior. Coincidental events include divorce, changing of living situation, changes in school or work schedule, physical injury, changes in a setting such as construction, changes in coworkers or staffing, and many others. Coincidental events share the characteristic that their behavioral impact is expected to be a *function of particular dates*. Controlling for coincidental events requires attention to the specific dates on which events occur.

Each of these three types of threats point us to distinct dimensions of the lag between phase changes that must be controlled for in order to achieve experimental control: for maturation, we control for elapsed time (e.g., days); for testing and session experience, we must be concerned with the number of sessions; and for coincidental events, we must be concerned with the specific time periods (i.e., calendar dates) of the study. Table 1 summarizes these threats to internal validity and the dimension of lag necessary to control for each.

**Table 1:** Primary threats to internal validity in multiple baseline designs

Threat	Alternative explanation (Source of effect)	Functional definition	Dimension of lag
Maturation	Unidentified biological events and environmental interactions	Behavioral changes that accumulate across days	Number of days
Testing & session experience	Any features of sessions, other than the IV (e.g., baseline procedures)	Behavioral changes that accumulate across sessions	Number of sessions
Coincidental events (i.e., History)	Extraexperimental events that coincide with the introduction of the independent variable	Behavioral changes that begin on a specific date	Specific dates

Multiple baseline designs can rigorously control these threats to internal validity. The process begins with a simple baseline-treatment (AB) comparison—a change from baseline to treatment within a single tier. If the baseline phase provides sufficiently stable data to support a strong prediction of the subsequent data path and the data path prediction is contradicted by the actual data after the introduction of the independent variable, this provides some suggestion that the independent variable may have been the cause of the change—a potential treatment effect. We use the term *potential treatment effect* to emphasize that the evidence provided by this single AB within-tier comparison is not sufficient to draw a strong causal conclusion because many threats to internal validity may be plausible alternative explanations for the data patterns. That is, experimental control has not been convincingly demonstrated. Adding multiple tiers to the design allows for two types of additional comparisons to be used to evaluate, and perhaps rule out, these threats: (1) replications of baseline-treatment comparisons *within* subsequent tiers (i.e., horizontal analysis), and (2) comparisons *across* tiers (i.e., vertical analysis).

The within-tier analysis seeks replication of these potential treatment effects in additional tiers of the design. If this pattern—a clear prediction from baseline being contradicted when and only when the independent variable is introduced—can be replicated across additional tiers of the multiple baseline, then the evidence of a treatment effect is incrementally strengthened. Although it is plausible that an extraneous variable’s influence could coincide with one phase change, it is less plausible that such a coincidence would occur twice, and even less plausible that it would occur three times. Additional replications further reduce the plausibility of extraneous variables causing change at approximately the same time that the independent variable is applied to each tier. Any alternative explanation of this pattern of results would have to posit an alternative set of causes that could plausibly result in changes in the dependent variable in this specific pattern across the multiple tiers. A critical requirement of the within-tier analysis is that no single extraneous event could plausibly cause the observed changes in multiple tiers. If this requirement is not met and a single extraneous event could explain the pattern of data in multiple tiers, then replications of the within-tier comparison do not rule out threats to internal validity as strongly. This critical requirement is mainly addressed by the lag between phase changes in successive phases. The time lag must be sufficiently long so that no single event could produce potential treatment effects in more than one tier. Other design features that contribute to the isolation of tiers such that any single extraneous variable is unlikely to contact multiple tiers can also strengthen the independence of tiers.

The across-tier comparison is an additional basis for evaluating alternative explanations. Data from the treatment phase in one tier can be compared to corresponding baseline data in another tier. If a potential treatment effect is observed in the treated tier but a change in the dependent variable is also observed in corresponding sessions in a tier that is still in baseline, this provides evidence that an extraneous variable may have caused both changes. This pattern seriously weakens the argument that the independent variable was responsible for the change in the treated tier. On the other hand, if we see a change in a treated tier and no change in untreated tiers, does this constitute strong evidence to rule out threats to internal validity? This argument rests on the assumptions that any extraneous variable that affects one tier will (1) contact all tiers and (2) have a similar effect on all tiers. If these assumptions are not valid, then it would be possible to observe stable baselines in untreated tiers even though the change in the treated tier was a result of an extraneous variable. In this case, the across-tier comparison would give the false appearance of strong internal validity. It is clear that we cannot claim that these assumptions are *always* valid for multiple baseline designs. The details of situations in which this across-tier comparison is valid for ruling out threats to internal validity are more complex than they may appear. We will explore these issues extensively after we sketch the historical development of multiple baseline designs and criticisms of nonconcurrent multiple baselines.

## Early Literature and Development of Methodology of Concurrent Multiple Baseline Designs

The multiple baseline design was initially described by Baer et al. in their classic 1968 article that defined applied behavior analysis. The authors discuss “two designs commonly used to demonstrate reliable control of an important behavior change” (p. 94). The first is the reversal design and the authors describe the important applied limitation with this design—situations in which reversals are not possible or feasible in applied settings. They then describe the “multiple baseline technique” (p. 94) and two types of comparisons that contribute to its experimental control. First, in the replicated within-tier comparison, each tier of the design is exposed to the treatment at a different point in time. After implementing the treatment for the first tier, they say, “rather than reversing the just produced change, he instead applies the experimental variable to one of the other as yet unchanged responses. If it changes at that point, evidence is accruing that the experimental variable is indeed effective, and that the prior change was not simply a matter of coincidence” (p. 94). Second, in a remarkably understated reference to the across-tier comparison, Baer et al. write that after implementing the treatment in an initial tier, the experimenter “perhaps notes little or no change in the other baselines” (p. 94). They do not elaborate on the importance of this type of comparison.

Hersen and Barlow’s (1976) textbook appears to be the first complete description of the multiple baseline design with many of the ideas about experimental control that are current to this day. They describe the control afforded by the design: “The experimenter is assured that his treatment variable is effective when a change in rate appears after its application while the rate of concurrent (untreated) behaviors remains relatively constant” (p. 226). Later they present an overall evaluation of the strength of multiple baseline designs, attributing its primary weakness to its reliance on the across-tier comparison, “The multiple baseline design is considerably weaker than the withdrawal design as the controlling effects of the treatment on each of the target behaviors is not directly demonstrated . . . the effects of the treatment variable are inferred from the untreated behaviors” (p. 227). In this highly influential early textbook on SCD, Hersen and Barlow describe only the across-tier analysis and fail to mention replicated within-tier comparisons. This has at least two effects: first, the multiple baseline is seen as weaker than the withdrawal design because of this dependence on the across-tier analysis; and second, when nonconcurrent multiple baseline designs are introduced years later, their rigor will be understood by many methodologists in terms of control by across-tier comparisons only, without consideration of replicated within-tier comparisons. These views of multiple baseline designs have been carried through into much of the single-case methodological literature and textbooks to the current day. It is interesting that this emphasis on across-tier comparisons is the opposite of that evident in Baer et al. (1968) who emphasized the replicated within-tier comparison.

Kazdin and Kopel (1975) parallel much of Hersen and Barlow’s (1976) commentary^3^ but they also point out an apparent contradiction in the assumptions about behavior on which the multiple baseline design is built. In order to demonstrate experimental control, the researcher makes two paradoxical assumptions. First, the design assumes that treatment effects will be tier-specific and not spread to untreated tiers. If an effective treatment were to have a broad impact on multiple tiers, the logic of the design would be to falsely attribute these effects to possible extraneous variables. Second, the across-tier comparison assumes that extraneous variables will affect multiple tiers similarly. If an extraneous variable were to have a tier-specific effect, it would be falsely interpreted as a treatment effect. This comparison can reveal the influence of an extraneous variable only if it causes a change in several tiers at about the same time. Thus, to demonstrate experimental control, the effects of the independent variable *must not generalize*; and to detect an extraneous variable through the across-tier comparison, the effects of that extraneous variable *must generalize*. As Kazdin and Kopel point out, it is clearly possible for treatments to have broad effects on multiple tiers and for extraneous variables to have narrow effects on a specific tier. This is a significant problem for the across-tier comparison because its logic is dependent on these two assumptions.

## Introducing the Nonconcurrent Multiple Baseline Design

The concurrent multiple baseline design opened up many new opportunities to conduct applied research in contexts that were not amenable to other SCDs. However, researchers in clinical, educational, and other applied settings recognized that they could expand research much further if the tiers of a multiple baseline could be conducted as they became available sequentially rather than simultaneously. Two articles published in 1981 described and advocated the use of nonconcurrent multiple baseline designs (Hayes, 1981; Watson & Workman, 1981).

Watson and Workman (1981) noted that “the requirement that observations be taken concurrently clearly poses problems for researchers in applied settings (e.g., schools, mental health centers), since clients with the same target behavior may only infrequently be referred at the same point in time” (p. 257). Watson and Workman described a nonconcurrent multiple baseline design in which participants could be begin a study as they became known to the researcher. The authors argue that like the concurrent multiple baseline design, the nonconcurrent form can rule out coincidental events (i.e., history) as a threat to internal validity and that experimental control can be established by the replication of the within-tier comparison with phase changes offset relative to the beginning of baseline. They do not mention the across-tier comparison, presumably because they believe that this analysis is not necessary to establish experimental control. Watson and Workman did not explicitly address threats to internal validity other than coincidental events.

Independent from Watson and Workman (1981), Hayes (1981) published a lengthy article introducing SCDs to clinical psychologists and made the point that these designs are well-suited to conducting research in clinical practice. When he turned to multiple baseline designs, Hayes argued that AB designs are natural to clinic work and that forming a multiple baseline can consist of collecting several AB replications, which would “inevitably have differing lengths of baseline” (i.e., a nonconcurrent multiple baseline; p. 206). He acknowledged that earlier authors had stated that multiple baselines must be concurrent and he noted that in a nonconcurrent multiple baseline the across-tier comparison could not reveal coincidental events. Hayes argued that “fortunately the logic of the strategy does not really require” (p. 206) an across-tier comparison because the within-tier comparison rules out these threats. Thus, both of the articles introducing nonconcurrent multiple baselines made explicit arguments that replicated within-tier comparisons are sufficient to address the threat of coincidental events.

## The Primary Methodological Criticism of Nonconcurrent Multiple Baselines: Across-Tier Comparisons

The current SCD methodological literature and most SCD textbooks claim that because the tiers of nonconcurrent multiple baseline are not synchronized in real time they have a diminished capacity to control for extraneous variables, in particular coincidental events (e.g., Carr, 2005; Gast et al., 2018; Harvey et al., 2004; Johnston et al., 2020). For example, Gast et al. (2018) state:

Confidence that maturation and history [coincidental events] threats are under control is based on observing (a) an immediate change in the dependent variable upon introduction of the independent variable, and (b) baseline (or probe) condition levels remaining stable while other tiers are exposed to the intervention. Without the latter you cannot conclude, with confidence, that the intervention alone is responsible for observed behavior changes since baseline (or probe) data are not concurrently collected on all tiers from the start of the investigation. Only through repeated measurement across all tiers from the start of a study can you be confident that maturation and history threats are not influencing observed outcomes. (p. 325)

Johnston et al. (2020) write:

Compared to its concurrent multiple baseline design sibling, a non-concurrent arrangement is inherently weaker . . . because a non-concurrent design does not allow any AB comparisons across baselines, it omits the opportunity to see if responding under the control condition changes when the treatment condition is implemented in the other baseline. (p. 365)

Barlow et al. (2009) state:

Of course, the major problem with this [nonconcurrent multiple baseline] strategy is that the control for history (i.e., the ability to assess subjects concurrently) is greatly diminished. Therefore, we view this approach as less desirable than the standard multiple baseline design across subjects and suggest that it should be employed only when the standard approach is not feasible. (pp. 234–235)

Although the claims that nonconcurrent multiple baseline designs are weaker than concurrent multiple baselines, especially with respect to threats of coincidental events, are nearly universal in the current literature, none of these authors acknowledge or address, the arguments made by Watson and Workman (1981) and Hayes (1981) in support of these designs. They never raise the question of whether replicated within-tier comparisons are sufficient to rule out threats to internal validity and establish experimental control. Having identified the criticisms of nonconcurrent multiple baseline designs, we now turn to a detailed analysis of threats to internal validity and features that can control these threats.

## Analysis of Control and Threats

In this section, we examine how within- and across-tier comparisons may support (or fail to support), internal validity in concurrent and nonconcurrent multiple baseline designs. We examine how these comparisons address maturation, testing and session experience, and coincidental events.

### Maturation

Concurrent and nonconcurrent multiple baseline designs address maturation in virtually identical ways through both within- and across-tier comparisons. For both types of comparisons, addressing maturation begins with an AB contrast in a single tier. If, in the initial tier, a pattern of stable baseline data is followed by a distinct change soon after the phase change, this constitutes a potential treatment effect. However, it does not rule out maturation as an alternative explanation of the change in behavior. Although many maturational changes are gradual, more sudden changes are possible. Further, if the potential treatment effect is more gradual (as one might expect from an educational intervention on a complex skill), maturational changes may be impossible to distinguish from treatment effects. The replicated within-tier analysis looks to patterns of results within the other tiers. If the pattern of change shortly after implementation of the treatment is replicated in the other tiers after differing lengths of time in baseline (i.e., different amounts of maturation), maturation becomes increasingly implausible as an alternative explanation. For example, it is implausible that the effects of maturation would coincide with a phase change after 5 days in one tier, after 10 days in a second tier, and after 15 days in a third. Thus, for any multiple baseline design to address the threat of maturation, it must show changes in multiple tiers after substantially differing numbers of days in baseline. The lag between phase changes must be long enough that maturation over any single amount of time cannot explain the results in multiple tiers.

The across-tier comparison provides another possible source of control for maturation. This comparison may reveal a likely maturation effect. If we observe a potential treatment effect in one tier and corresponding changes in untreated tiers after similar amounts of time (i.e., number of days), maturation becomes a more plausible alternative explanation of the initial potential treatment effect. On the other hand, if we observe that one tier shows a change whereas other tiers that have been observed for similar amounts of time do not show similar changes, this may reduce the plausibility of the maturation threat. The assumption that maturation contacted all tiers is strong—participants were all exposed to maturational variables (i.e., unidentified biological events and environmental interactions) for the same amount of time. The assumption that all tiers respond similarly to maturation may be somewhat more problematic. A given period of maturation may affect various participants, various behaviors, or behaviors in various settings in different ways. For example, for a child who is on the cusp of walking, a month of exposure to maturational variables may result in a significant improvement in walking, but much less change in fine motor skills. (Similar arguments can be made for comparisons across settings, persons, and other variables that might define tiers.) The point is that although the across-tier comparison *may* reveal a maturation effect, there are also circumstances in which it *may fail* to do so. Thus, although the across-tier analysis does provide a test of the maturation threat, a lack of change in untreated tiers cannot definitively rule it out.

To summarize, the replicated within-tier analysis with sufficient lag can rigorously control for the threat of maturation. The across-tier analysis can provide an additional set of comparisons that may reveal a maturation effect, but it is not a conclusive test. Neither the within-tier comparison, nor the across-tier comparison depends on the tiers being conducted simultaneously; both types of comparisons only require that phase changes occur after substantially different amounts of time since the beginning of baseline—that is, each tier is exposed to different amounts of maturation (i.e., days) prior to the phase change. As a result, concurrent and nonconcurrent designs are virtually identical in their control for maturation threats. Concurrence is not necessary to detect and control for maturation.

### Testing and Session Experience

In both forms of multiple baseline designs, a potential treatment effect in the first tier would be vulnerable to the threat that the changes in data could be a result of testing or session experience. However, if this within-tier pattern is replicated in multiple tiers after differing numbers of baseline sessions, this threat becomes increasingly implausible. An alternative explanation would have to suggest, for example, that in one tier, experience with 5 baseline sessions produced an effect coincident with the phase change; in a second tier, 10 baseline sessions had this effect, again coinciding with the phase change; and in a third tier, 15 baseline sessions produced this kind of change and happened to correlate with the phase change. Thus, a multiple baseline with phase changes sufficiently lagged (in terms of number of sessions) provides rigorous control for this threat.

Both concurrent and nonconcurrent multiple baseline designs also afford the same across-tier comparison; both can show a potential treatment effect after a certain number of baseline sessions in one tier and a lack of effect after that same number of sessions in another tier. We can strongly argue that all tiers contact testing and session experience during baseline because we schedule and conduct these sessions. However, an across-tier comparison is not definitive because testing or session experience could affect the tiers differently. For example, in a multiple baseline across settings, the settings could present somewhat different demands. If session experience exerted a small degree of influence on the DV, an effect might be observed in settings where the behavior is more likely, but not in settings where the behavior is less likely. So, similar to maturation, the across-tier comparison is sometimes able to reveal effects of testing and session experience, but it may fail to do so in some circumstances.

In both within- and across-tier comparisons, the dates on which the sessions took place are not relevant to the effects of testing and session experience. Ten sessions of baseline would be expected to have similar effects whether they occur in January or June. Therefore, concurrent and nonconcurrent designs are virtually identical in control for testing and session experience.

### Coincidental Events

The nature of control for coincidental events (i.e., history) provided by the within-tier comparison in both concurrent and nonconcurrent multiple baseline designs is relatively straightforward. A potential treatment effect in any single tier could plausibly be explained as a result of a coincidental event. However, each replication of the possible treatment effect that takes place at a substantially distinct calendar date reduces the plausibility of this threat. Each replication requires an assumption of a separate event coinciding with a distinct phase change. This control assumes that the replications are sufficiently offset in real time (e.g., calendar days) to ensure that a single coincidental event could not plausibly cause the effects observed in multiple tiers.

The strength of this control is a function of our certainty that no single coincidental event could have caused more than one change in the dependent variable. This certainty is increased by isolation of tiers in time and other dimensions. The dimension of time is recognized in the requirement that phase changes be lagged in real time—that is, the date on which the phase changes are made. In general, a longer lag is better because it reduces the chance that an event could impact multiple tiers. If a nonconcurrent multiple baseline has a long lag in real time between phase changes (e.g., weeks or months), this may provide stronger control than a design with a lag of one or several days. Thus, to the degree that nonconcurrent designs support longer lags between phases changes than concurrent designs, they may support stronger control of the threat of coincidental events through replicated within-tier comparisons. The within-tier comparison may be further strengthened by increasing independence of the tier in other dimensions. If each tier of a multiple baseline represents a different participant in a different environment (e.g., school versus clinic) located in a different city, this would further reduce the chance that any single event or pattern of events could have contacted the participants coincident with the phase changes. The logic of replicated within-tier analysis applies equally to concurrent and nonconcurrent designs.

The across-tier analysis of coincidental events is the main way that concurrent and nonconcurrent multiple baselines differ. According to conventional wisdom, concurrent multiple baselines are superior because they allow for across-tier comparisons that can rule out coincidental events. If a potential treatment effect is seen in one tier and on the same day there is no change in other tiers, this is taken as strong evidence that the potential treatment effect was not a result of a coincidental event, because a coincidental event would have had an effect on all tiers. Nonconcurrent multiple baseline designs, however, do not afford this comparison. If a potential treatment effect is seen in one tier, the researcher cannot refer to data from the same day in an untreated tier because the tiers are not synchronized in real time and may not even overlap in real time. This has been the sharpest point of criticism of nonconcurrent multiple baselines. However, critics of nonconcurrent designs have rarely (1) made a thorough and critical analysis of the potential weaknesses of across-tier comparisons in concurrent multiple baselines, or (2) evaluated the degree of experimental control that can be demonstrated by replicated within-tier comparisons.

As we mentioned above, across-tier comparisons require the assumptions that coincidental events will (1) contact and (2) have similar effects on all tiers of the design. To understand the ability of concurrent designs to meet these assumptions we must distinguish different types of coincidental events based on the scope of their effects. A coincidental event may contact a single unit of analysis (e.g., one of four participants) or multiple units (e.g., all participants). Events that contact a single participant may be termed *participant-level*. Examples could include family events, illness, changed social interactions (e.g., breaking up with a partner), losing or gaining access to a social service program, etc. These coincidental events would contact all tiers of a multiple baseline that include this individual participant, but not tiers that do not involve this participant. In a concurrent multiple baseline that involves a single participant across settings, behaviors, antecedent stimuli etc., this kind of event would be expected to contact all tiers. Thus, the assumption that the coincidental event contacts all tiers would be valid and the across-tier analysis might reveal the effects of this sort of event. However, *in a concurrent multiple baseline across participants, participant-level events contact only a single tier (participant)—the coincidental event would not contact other tiers (participants)—we might say that the across-tier analysis is inherently insensitive to detecting this kind of event.* This insensitivity is not due to poor experimental design or implementation, it is built in to the nature of multiple baseline designs across participants. Each tier involves a unique participant and there is a class of coincidental events that contact a single participant.

Likewise, *setting-level* coincidental events are those that contact a single setting. Potential setting-level events include staffing changes in classroom, redecoration or renovation of the physical environment, and changes in the composition of the peer group in a classroom, group home, or worksite. These events would contact all tiers of a MB that take place in that single setting, but not tiers in other settings. In concurrent multiple baseline across participants, behaviors, or stimulus materials that take place in a single setting, this kind of event would contact all the tiers of the multiple baseline. In this case, the effects of this kind of event could be revealed through the across-tier comparison of participants or behaviors that have not been exposed to the independent variable. However, in a concurrent multiple baseline *across settings* a setting-level event would contact only a single tier—the design would be inherently insensitive to these coincidental events.

*In general, in a concurrent multiple baseline design across any factor, the across-tier analysis is inherently insensitive to coincidental events that are limited to a single tier of that factor. Under these conditions, the experimental rigor of concurrent multiple baselines is identical to nonconcurrent multiple baselines; coincidental events that contact a single tier cannot be detected by an across-tier analysis.* The problem of tier-specific coincidental events can be reduced by selecting tiers that differ on only a single factor (e.g., participants, settings, behaviors) and are as similar as possible on that factor. For example, there is less room for participant-level coincidental events if all participants reside in a single group home than if they reside in different group homes in different states. However, as Hayes (1985) pointed out, even with the most rigorous care in experimental design, we can never give two individuals the same experiences outside of our experimental sessions. Likewise, in a multiple baseline across settings, selecting settings that tend to share extraneous events would make the across-tier analysis more powerful than would selecting settings that share few common events. For example, two rooms in the same treatment center would share more coincidental events than a room in a treatment center and another room at home. However, we can never ensure that any two contexts or any two session times are not subject to unique events during the study. The bottom line is that the experimenter can never know whether a coincidental event has contacted only a single tier of a concurrent multiple baseline and, therefore, whether it is possible for the across-tier comparison to detect this threat.

Further, for the across-tier comparison to detect the influence of a coincidental event, that event must not only *contact* multiple tiers, it must *cause similar changes* in the dependent measure across multiple tiers. It is possible that a coincidental event may be present for all tiers but have different effects on different tiers. As Kazdin and Kopel (1975) pointed out, multiple baseline designs require that the effects of the independent variable must have tier-specific effects, yet the across-tier analysis requires that extraneous variables must not have tier-specific effects. For example, in a multiple baseline across participants, all the residents of a group home may contact peanut butter and jelly sandwiches for lunch but this change may disrupt the behavior of residents with a mild peanut allergy, but not other residents. Or in a multiple baseline across settings that are assessed at different times of the day, a socially challenging event such as an increase in daily bullying on a morning bus ride could disrupt the target behavior of a participant for the first hour of the day, but have reduced effects thereafter. A multiple baseline design with tiers conducted at different times during each day could show disruption due to this coincidental event in the tier assessed early in the day but not in tiers that are assessed later in the day. Such events might be said to *contact* all tiers, but *affect* only one of them.

There is ample empirical evidence of differential impact of variables across tiers. Every multiple baseline design in which potential treatment effects are observed in some but not all tiers demonstrates that tiers are not always equally sensitive to interventions. And researchers generally design and implement interventions, select tiers, and employ measures that will likely show consistent treatment effects. Coincidental events might be expected to be more variable in their effect than interventions that are designed to have consistent effects. This assumption was initially identified by Kazdin and Kopel in 1975, but its implications for the rigor of the across-tier comparison have rarely been discussed since that time. These observations lead us to the conclusion that neither of the critical assumptions that coincidental events will (1) contact and (2) have similar impact on all tiers can be assumed to be valid. *If either of these assumptions are not valid for a coincidental event, then the presence and function of that event would not be revealed by the across-tier analysis*. We are not pointing to flaws in execution of the design; we are pointing to inherent weaknesses. Poor execution can certainly worsen these problems, but good execution cannot eliminate them. The across-tier comparison of concurrent multiple baseline designs is less certain and definitive than it may appear. Although the across-tier comparison may detect some coincidental events; it cannot be assumed to detect them all. Further, it is impossible to know how many events, which events, or the severity of the events that are missed by an across-tier comparison. By nature, undetected events are unknown. A researcher who puts great confidence in the across-tier comparison could falsely reject the idea that coincidental events were the cause of observed effects.

### A Second Methodological Criticism of Nonconcurrent Designs: Prediction, Verification, Replication

Cooper et al. (2020) make a somewhat different methodological criticism of nonconcurrent multiple baseline designs. Throughout their discussion of SCD, these authors describe experimental control in terms of three processes: prediction, verification, and replication. In the case of multiple baseline designs, a stable baseline supports a strong *prediction* that the data path would continue on the same trajectory in the absence of an effective treatment; these predictions are said to be *verified* by observing no change in trajectories of data in other tiers that are not subjected to treatment; and *replication* is demonstrated when a treatment effect is seen in multiple tiers. They argue that because nonconcurrent multiple baseline designs lack an across-tier comparison in real time (the criticism described above), they cannot *verify* the prediction of the behavior pattern in the absences of intervention. They state,

the nonconcurrent multiple baseline across participants design is inherently weaker than other multiple baseline design variations. Although the design entails two of the three elements of baseline logic—prediction and replication—the absence of concurrent baseline measures precludes the verification of [the prediction]. (p. 206)

Carr (2005) invokes this prediction, verification, and replication logic, and concludes, “The nonconcurrent MB design only controls for threats associated with maturation/exposure; it does not control for historical [coincidental events] threats to internal validity, as does a concurrent MB design” (p. 220).

As we argued above, the observation of no change in an untreated tier is not strong evidence against a coincidental event affecting the treated tier. That is, it is not strong evidence verifying the prediction of no change in the initial tier in the absence of an intervention. However, this kind of support is not necessary: lagged replications of baseline predictions being contradicted by data in the treatment phase provide strong control for all of these threats to internal validity. Perhaps a more general and powerful triad of processes that support demonstration of experimental control would be prediction, contradiction, and replication. This would draw attention to the relationship between the prediction from baseline and the (possible) contradiction of that prediction by the obtained treatment-phase data, and the replication of this prediction-contradiction pair in subsequent tiers.

#### Number of Tiers Required to Demonstrate Experimental Control

Throughout this article we have referred to the importance of replicating within-tier comparisons, emphasizing the idea that tiers must be arranged with sufficient lag in phase changes so that specific threats to internal validity are logically ruled out. This raises the question of how many replications are necessary to establish internal validity. The functional answer to this question is that there must be sufficient tiers so that none of the threats to internal validity are plausible explanations for the pattern of effects across the set of tiers. This statement, of course, fails to satisfy the operational desire for a specific number of tiers that accomplishes this function. Because experimental circumstances and design elements vary so greatly, no universal answer can be given. We can identify at least three general categories of issues that influence the number of tiers required to render threats implausible: challenges associated with the phenomena under study, experimental design features, and data analysis issues.

First, studies differ with respect to the experimental challenges imposed by the phenomena under study. Features of the target behaviors, participants, measurement, and so forth can make threats to internal validity more or less likely. For example, in a study of language skills in typically developing 3-year-old children, maturation would be a particular concern. It would be an even greater concern if the treatment were an instructional program that requires several weeks or months to implement. Testing and session exposure may be particularly troublesome in a study that requires taking the participant to an unusual location and exposing them to unusual assessment situations in order to obtain baseline data. A study may be at heightened risk of coincidental events if the target behavior is particularly sensitive to events in the environment that are uncontrolled by the experimenter. Any of these types of circumstances may require additional tiers in order to clearly address threats to internal validity.

Second, as we have discussed above, the amount of lag between phase changes (in terms of sessions in baseline, days in baseline, and elapsed days) is the primary design feature that reduces the plausibility of any single threat accounting for changes in multiple tiers, and thereby threatening the internal validity of the design as a whole. In addition, functionally isolating tiers (e.g., across settings) such that they are highly unlikely to be subjected to the same instances of a threat can also contribute to this goal. Longer lags and more isolated tiers can reduce the number of tiers necessary to render extraneous variables implausible explanations of results.

Third, patterns of results influence the number of tiers needed to yield definitive conclusions. Data analysis issues concern two closely related questions: (1) Was there a change in data patterns after the phase change? and (2) Was any change the result of the independent variable? To answer the first question, the one must distinguish signal (systematic change) from noise (unsystematic variance). This has been the topic of important recent methodological research, including studies of the interobserver reliability of expert judgements of changes seen in published multiple baseline designs (Wolfe et al., 2016) and use of simulated data to test Type I and II error rates when judgements of experimental control are made based on different numbers of tiers (Lanovaz & Turgeon, 2020). The present article is focused on the second question—whether systematic changes in data can be attributed to the treatment. This question cannot be addressed by data analysis alone; any pattern of data, no matter how dramatic, could be a result of an extraneous variable if the experimental design features are not properly arranged. Addressing the second question requires data analysis that is informed by the specifics of the study. Still, for a given study, the results influence the number to tiers required in a rigorous multiple baseline design. When changes in data occur immediately after the phase change, are large in magnitude, and are consistent across tiers, threats to internal validity tend to be less plausible explanations of the data patterns, and fewer tiers would be required to rule them out.

To offer some guidance, we believe that under ideal conditions—adequate lags between phase changes, circumstances that do not suggest that threats are particularly likely, and clear results across tiers—three tiers in a multiple baseline can provide strong control against threats to internal validity. This is consistent with the judgements made by numerous existing standards and recommendations (e.g., Gast et al., 2018; Horner et al., 2005; Kazdin, 2021; Kratochwill et al., 2013). When conditions are less ideal, additional tiers may be necessary. In the end, judgments about the plausibility of threats and number of tiers needed must be made by researchers, editors, and critical readers of research.

## Conclusions and Recommendations

Throughout this article we have argued that controlling for the three main threats to internal validity—maturation, testing and session experience, and coincidental events—in multiple baseline designs requires attention to three distinct dimensions of lag of phase changes across tiers. Controlling for maturation requires baseline phases of distinctly different temporal durations (i.e., number of days); controlling for testing and session experience requires baseline phases of substantially different number of sessions; and controlling for coincidental events requires phase changes on sufficiently offset calendar dates. All three of these dimensions of lag are necessary to rigorously control for commonly recognized threats to internal validity and establish experimental control. Therefore, we believe that these features should be explicitly included in the definition of multiple baseline designs. We recommend that *multiple baseline design* be defined as *a single-case experimental design that evaluates causal relations through multiple baseline-treatment comparisons with phase changes that are sufficiently offset in (1) real time (i.e., calendar date), (2) number of days in baseline, and (3) number of sessions in baseline*. This would align the definition with the critical features required to demonstrate experimental control and thereby allow strong causal statements based on multiple baseline designs. Without these dimensions of lag explicitly stated in the definition, we cannot claim that multiple baseline designs will necessarily include the features required to establish experimental control. The definition states that there must be *sufficient* lag between phase changes—this is not further specified because the amount of lag necessary to ensure that any single amount of maturation, number of sessions, or coincidental event could not cause changes in multiple tiers must be determined in the context of the particular study. An important question for researchers, reviewers, and readers of research is whether the amount of lag is *sufficient* for a specific study.

Recognizing these three dimensions of lag has implications for reporting multiple baseline designs. The vast majority of contemporary published multiple baseline designs describe the timing of phases in terms of sessions rather than days or dates. This provides clear information about the number of sessions that precede the phase change in each tier, and therefore constitutes a strong basis for controlling the threat of testing and session experience. However, current practice provides little or no direct information on either the temporal duration (e.g., number of days) of baseline nor the offset between phase changes in real time (i.e., number of calendar days between phase changes). These reports do not provide the information necessary to rigorously evaluate maturation or coincidental events. For example, phase changes in two consecutive tiers may be lagged by three sessions, but if one to three sessions are conducted per day, the baseline phases could include the same number of days (problem for controlling maturation) and the phase change could occur on the same day in both tiers (problem for controlling coincidental events). Under the proposed definition, such a study would not be considered a full-fledged multiple baseline. In order to meet the terms of the definition, and confirm the critical characteristics for controlling threats to internal validity, we recommend that all multiple baseline studies explicitly report, for each tier, the number of days and sessions in each phase, and the number of calendar days of phase change lag from the previous tier. This might be conveniently reported in the methods section or a small table in an appendix. This information would allow readers to evaluate the sufficiency of each dimension of lag given the specific characteristics of the particular study.

Based on the logic laid out in this article, we believe that the treats of maturation and testing and session experience are controlled equivalently in concurrent and nonconcurrent design. Further, for both types of multiple baselines, the threat of coincidental events should be evaluated primarily based on replicated within-tier comparisons. Any one tier may, at best, demonstrate a *potential* treatment effect; however, a set of three or more tiers may strongly address the threat of coincidental events and clearly demonstrate experimental control. The across-tier comparison is valuable primarily when it suggests the presence of a threat by showing a change in an untreated tier at approximately the same time (i.e., days, sessions, or dates) as a potential treatment effect. The lack of change in untreated tiers should be interpreted only as weak evidence supporting internal validity given the plausible alternative explanations of this lack of change.

This understanding of the primary role of replicated within-tier comparisons also implies that, when there is a trade-off, design options that improve control through the within-tier comparisons should take precedence over those that would improve control through across-tier comparisons. In particular, within-tier comparisons may be strengthened by isolating tiers from one another in ways that reduce the chance that any single coincidental event could coincide with a phase change in more than one tier (e.g., temporal separation). On the other hand, across-tier comparisons may be strengthened by arranging tiers to be as similar as possible so that they would be more likely to be exposed to the same coincidental events. Given this dilemma, priority should be given to optimizing the within-tier comparisons because this is the comparison that can confer stronger control. Thus, the additional temporal separation that is possible in a nonconcurrent design is a strength rather than a weakness in controlling for coincidental events. In addition, arranging tiers that are isolated in other dimensions (e.g., location, behaviors, participants) confers overall strength, not weakness, for addressing coincidental events.

With control for coincidental events in multiple baseline designs resting squarely on replicated within-tier comparisons, there is no basis for claiming that, in general, concurrent designs are methodologically stronger than nonconcurrent designs. Textbook authors, editors, and readers of research should consider nonconcurrent multiple baseline designs to be capable of supporting conclusions every bit as strong as those from concurrent designs. The issue of concurrence of tiers should be considered along with many other design variations that can be manipulated to create a design that fits the particular experimental challenges of a particular study. Instead, the idea that lag across phase changes includes three important dimensions and that these lags are critical for establishing experimental control and justifying strong causal conclusions should be elevated in importance.

## Footnotes

1 Other threats to internal validity such as (1) ambiguous temporal precedence, (2) selection, (3) regression, (4) attrition, and (5) instrumentation are addressed primarily through other design features. For example, instrumentation is addressed primarily through observer training, calibration, and IOA.

2 Elapsed time does not directly cause maturational changes in behavior. Rather, the passage of time allows for more opportunities for participants to interact with their environment—leading to maturational changes. We use *function of elapsed time* descriptively rather than causally.

3 Although publication dates would suggest that Kazdin and Kopel (1975) was published before Hersen and Barlow (1976), Kazdin and Kopel cite Hersen and Barlow, and not the other way around.
